# Neuroanatomical predictors of complex skill acquisition during video game training

**DOI:** 10.3389/fnins.2022.834954

**Published:** 2022-07-22

**Authors:** Anna Kovbasiuk, Paulina Lewandowska, Aneta Brzezicka, Natalia Kowalczyk-Grębska

**Affiliations:** ^1^Neurocognitive Research Center, Institute of Psychology, SWPS University of Social Sciences and Humanities, Warsaw, Poland; ^2^Department of Management in Networked and Digital Societies, Kozminski University, Warsaw, Poland; ^3^Institute of Psychology, Jagiellonian University, Kraków, Poland

**Keywords:** video game training, MRI, predispositions, individual differences, complex skill acquisition, performance enhancement, Scaffolding Theory of Aging and Cognition

## Abstract

It is known that the outcomes of complex video game (VG) skill acquisition are correlated with individual differences in demographic and behavioral variables, such as age, intelligence and visual attention. However, empirical studies of the relationship between neuroanatomical features and success in VG training have been few and far between. The present review summarizes existing literature on gray matter (GM) and white matter correlates of complex VG skill acquisition as well as explores its relationship with neuroplasticity. In particular, since age can be an important factor in the acquisition of new cognitive skills, we present studies that compare different age groups (young and old adults). Our review reveals that GM in subcortical brain areas predicts complex VG learning outcomes in young subjects, whereas in older subjects the same is true of cortical frontal areas. This may be linked to age-related compensatory mechanisms in the frontal areas, as proposed by The Scaffolding Theory of Aging and Cognition. In the case of plasticity, there is no such relationship – in the group of younger and older adults there are changes after training in both cortical and subcortical areas. We also summarize best practices in research on predictors of VG training performance and outline promising areas of research in the study of complex video game skill acquisition.

## Introduction

Understanding skill acquisition in cognitive training can be important in many different areas – from education through professional training to neurorehabilitation. Complex skill training has an important advantage over simple behavioral interventions, since the findings of studies using the former generalize better to tasks performed in real life, outside the laboratory (Boot, 2015; Boot et al., 2017). The complex tasks that have been shown to exert a long-term effect on human brain anatomy include juggling (Draganski et al., 2004), musical training (Lappe et al., 2011), balancing (Taubert et al., 2010), dancing (Rehfeld et al., 2018), as well as video games (VGs) (Momi et al., 2018).

Video games are classified as complex tasks because they engage multiple cognitive faculties simultaneously, impose complex rules and decision-making conditions, require prolonged practice to master specific skills and include motivational elements, which heighten engagement (Boot, 2015; Boot et al., 2017).

Moreover, in comparison to other activities, VGs are widely popular and available for various segments of the population. Global market reports confirm the growing interest in gaming and predict that the number of players will increase in 2021 to 3 billion gamers (+5.4% from the previous year) (Newzoo, 2020).

Video games are also widely used by people with disabilities (46 million players in the US alone) (ESA.com, 2020). Studies have shown that VG interventions are a promising tool in therapy, in rehabilitation of patients with cognitive deficits, as well as in neuropsychological assessment (Suenderhauf et al., 2016; Colder Carras et al., 2017). For example, according to (Han et al., 2008), patients with schizophrenia who played internet video games exhibited reduced psychotic symptoms and extrapyramidal side effects, whereas other researchers (Suenderhauf et al., 2016; Colder Carras et al., 2017) claim that VGs can be effective in reducing symptoms in patients with psychosis. These findings provide evidence that VG training induces psychological changes, which can be linked to neural plasticity in the group of patients.

There is also some evidence that VGs can be effective in supporting cognitive functions in the elderly (e.g., Basak et al., 2011). Moreover, researchers investigating complex skill acquisition concentrated not only on training as compensation for loss or deterioration of brain functions, but also on its effects on brain plasticity in healthy young adults (e.g., Erickson et al., 2010; Momi et al., 2018, 2019).

The various ways in which playing VGs may affect cognition have been the topic of many studies, some suggest beneficial effects of VGs on human behavior (Connolly et al., 2012; Oei and Patterson, 2014; Shams et al., 2015; Wang et al., 2016; Bediou et al., 2018; Kühn et al., 2019; Choi et al., 2020) and the brain (Shams et al., 2015; Suenderhauf et al., 2016; Bavelier and Green, 2019; Brilliant et al., 2019; Kühn et al., 2019), others fail to find any impact (Bisoglio et al., 2014; Sala et al., 2018), or even show that playing games may harm cognitive functions (Anderson et al., 2010; Greitemeyer and Mügge, 2014; John et al., 2019). Studies reveal that VG players (VGPs) in comparison to non-video game players (NVGPs) are better at attentional tasks (Dye et al., 2009; Wong and Chang, 2018), tracking faster objects, task switching and object rotation (Boot et al., 2008). Even extensive, over 20 h-long action VG training undergone by NVGPs did not compensate for these differences between them (Boot et al., 2008). Furthermore, both computer and mobile players outperform NVGPs at executive function tasks (Huang et al., 2017). Experts have higher scores also in visual short-term memory/working memory tests (Boot et al., 2008; Blacker and Curby, 2013; Colzato et al., 2013). This is also confirmed by self-reports of gamers among which 80% consider that VGs provide mental stimulation (ESA.com, 2020).

Cross-sectional studies correlating the amount of game practice and player scores in various domains provided important insights into cognitive and behavioral benefits of VGs (Green and Bavelier, 2003, 2007). Cross-sectional studies also linked VG practice to differences between the brains of players and non-players (Kühn and Gallinat, 2014). In particular, the gray matter (GM) of the entorhinal cortex, hippocampus and occipital areas – which are related to navigation, memory (Eichenbaum et al., 2007) and visual attention (Corbetta and Shulman, 2002) – was found to be correlated with the total amount of time spent playing VGs (Kühn and Gallinat, 2014).

Experimental studies with VG training intervention VGs revealed that due to their complexity and high cognitive demands, VGs enhance executive control in such tasks as switching, reasoning and visual short-term memory (Basak et al., 2008; Stern et al., 2011). This also results in transfer to untrained tasks. In the group of young VGP when compared to NVGPs researchers observed near-transfer to the tasks of monitoring and updating of working memory but no general gains (Colzato et al., 2013). Furthermore, in another study after action VG training in the group of younger subjects performance improvement only in tasks similar to the ones exercised by VG as elimination of attentional blink effect, improvement in keeping track of multiple objects simultaneously as well as cognitive control in filtering irrelevant stimuli were found (Oei and Patterson, 2013). In the group of older subjects there were far-transfer effects observed but they were dependent on the type of VG training participants completed. After the brain training VGs far transfer to everyday problem solving was found, whereas after non-commercial Space Fortress VG participants had both far transfer to problem solving and near transfer to working memory (Strenziok et al., 2014).

Experimental studies using VG training intervention in subjects who had not been VG players have also confirmed positive long-term influence of VGs on the human brain (e.g., Kühn et al., 2014, 2018; Momi et al., 2018, 2019; for review see Palaus et al., 2017; Brilliant et al., 2019). Significant increases in the GM of the prefrontal, temporal and parietal regions, the hippocampus (HIP) and cerebellum were observed in the group of younger and older adults (Colom et al., 2012; Kühn et al., 2014; Szabó et al., 2014; West et al., 2017). These regions are involved in numerous functions, including memory retrieval, attention and the executive function (Colom et al., 2012; Kühn et al., 2014; Szabó et al., 2014; West et al., 2017). Moreover, randomized experimental studies have also found heightened white matter (WM) integrity in the thalamus, lingual gyrus (LG), hippocampal cingulum and inferior longitudinal fasciculus (ILF) arter training in both groups (Colom et al., 2012; Strenziok et al., 2014).

However, some researchers failed to find any cognitive enhancement following training of non-video game players (Boot et al., 2008) or observed change only in certain domains in young subjects without general gains (Colzato et al., 2013). It has been suggested that the influence of personal traits and game mechanics on learning and success in VG skill acquisition should also be studied, since these factors may explain differences in the observed effects (Bavelier et al., 2011). A meta-analysis carried out by Wang et al. (2016) has shown that larger beneficial effects from VG training are more pronounced in younger than in older adults. Age, intelligence (Rabbitt et al., 1989) and baseline visual attention (Arthur et al., 1995) were also explored as predictors of VG training outcomes. Individual differences in neuroanatomy may explain the variability in VG learning outcomes (e.g., Erickson et al., 2010; Basak et al., 2011; Ray et al., 2017). Hence, in this review, we focus on the ways in which individual differences in brain anatomy (on gray and white matter levels) are linked to VG skill acquisition, and we explore the links between such variables as age and VG genre, and the results achieved. It has also been our aim to evaluate the available studies critically, paying attention to methodology and effect size, and to highlight prospects for further research on VG training success prediction. The present article should be of particular interest to three groups of readers: researchers working with professional e-sports players to optimize training regimes and selection for competition; practitioners aiming to create personalized neurorehabilitation tools and cognitive training methods, suitable both for patients and for healthy adults of any age; and scientists developing prediction models of performance enhancement in complex skill acquisition.

### Video games as a tool for the study of complex skill acquisition

Researchers studying cognitive enhancement use both commercially available and non-commercial (“serious”) VGs of various genres for training interventions (Choi et al., 2020). Researchers differentiate commercial and serious games based on the purposes for which they were created. Choi et al. (2020) defined serious games as those “developed for learning and changes of behavior in various areas,” as opposed to commercial VGs, created for entertainment. Space Fortress is a notable example of a serious VG, developed in order to study learning strategies and the effects of practice in complex task acquisition (Donchin, 1995). Space Fortress, although sometimes classified as an action VG (AVG) (e.g., Palaus et al., 2017), lacks some features of this genre (Bediou et al., 2018). According to a definition of action video games (AVGs) given in Bediou et al. (2018), such games may include a high perceptual and motor load, as well as a high degree of clutter and distraction – features missing from Space Fortress. Due to its purpose – research, rather than entertainment – Space Fortress can be less engaging for the player, in comparison to commercially available VGs.

As far as commercially available games are concerned, AVGs have often been found to improve cognitive functions. Though these games are sometimes associated with violence, the terms “violent game” and “action VG” are not synonymous, as pointed out by Bediou et al. (2018). According to a recent review by Choi et al. (2020), AVGs can be defined as games which have a “static physical locator connecting gaze and actions of players in the game environment.” While there is no universally accepted definition of AVGs, researchers commonly attribute a certain set of features to this genre.

According to the latest definition, given by Dale et al. (2020), these include:

“(1) a fast pace, meaning that players have to constantly react under time pressure, (2) the need for players to distribute their attention across the peripheral visual field to monitor for potential incoming threats, (3) the need for players to focus their attention when required, such as when shooting at an opponent, (4) the need to switch between these two states of attention (distributed/focused) upon demand, and (5) enough variability in the game, such as in the behavior of the opponents, to prevent full task automatization.”

An earlier, widely used definition of AVGs, proposed by Bavelier and Green (2019), listed features like the ones enumerated by Dale et al. (2020), but also added two other characteristics, viz. “(1) a high degree of perceptual and motor load, but also working memory, planning and goal setting (e.g., many items to keep track of simultaneously, many possible goal states that need to be constantly reevaluated, many motor plans that need to be executed rapidly) and (2) a high degree of clutter and distraction (i.e., items of interest are distributed among many non-target items).” According to Bavelier et al. (2012), AVGs facilitate quick acquisition of new tasks, helping the user develop the “learning to learn” skill. Animal studies have also shown the importance of a complex “enriched environment,” which may be responsible for stimulating neural plasticity in VG training (Bavelier et al., 2012). FPS, one of the most popular types of AVGs, is characterized by time pressure, attention demands for peripheral field of view, focus and the ability to switch attention on demand, variability and lack of automatization (Dale et al., 2020).

Strategy VGs (SVGs) – and real time strategy (RTS) games in particular – promote cognitive flexibility, switching across multiple activities and maintenance of sustained attention, and put high cognitive demands on the player (Glass et al., 2013; Vinyals et al., 2019). Players perform many tasks simultaneously, such as: managing resources, creating units, assigning units to battle (Dobrowolski et al., 2015), making real-time decisions and investigating novel strategies in a complex environment (Vinyals et al., 2019). As a result, RTS games can enhance visual and spatial skills (Dobrowolski et al., 2015; Kim et al., 2015). It is the second most popular genre in VG skill acquisition studies. FPS and RTS games differ in some important respects, with the latter involving a greater number of moving stimuli in the field of view, and an allocentric perspective (view from above), as opposed to FPS games, which adopt an egocentric perspective (first-person view) (Dobrowolski et al., 2015). A study comparing these two types of VGs has shown that players of RTS games are characterized by better abilities to track multiple objects and by greater processing speed (Dobrowolski et al., 2015). Interestingly, the environment of StarCraft II, an RTS VG, was used in artificial intelligence (AI) research – the DeepMind project – which also indicates its transferability and similarity to real-life tasks (Vinyals et al., 2019).

Another type of VGs used for cognitive enhancement comprises puzzles – usually classified as a subgenre of traditional games (TGs) – and simulation games (SGs), in which the player performs everyday activities or difficult tasks, such as flying or driving (Choi et al., 2020).

### Possible reasons for inconsistent results in studies of video game performance enhancement

Studies using VGs of various genres highlighted general cognitive, perceptual and emotional benefits of VG training interventions. These studies were summarized in multiple reviews and meta-analyses, which confirmed the effectiveness of VG interventions (Wang et al., 2016; Pallavicini et al., 2018). For instance, it was found in the metaanalysis of studies that AVGs have moderate influence on cognitive functions, and also that healthy young adults benefit more in comparison to older subjects (Wang et al., 2016). Younger adults in comparison to elderly benefited significantly more in overall cognition and processing speed/attention, whereas also there was a difference in visuospatial ability and executive function but insignificant (Wang et al., 2016).

Various theories have been proposed to explain both the changes in cognitive and brain functions caused by aging, and the compensatory mechanisms involved in this process. One such theory is the Scaffolding Theory of Aging and Cognition (STAC) (Park and Reuter-Lorenz, 2009). Authors of the theory proposed that brain structure changes and functional deterioration, which comes with aging, can trigger brain mechanisms to compensate for this loss of cognitive functions. How a person functions is determined by the interplay of those factors and positive mechanisms of plasticity, called “compensatory scaffolding” according to the theory. Therefore, it can be expected that young and older subjects will have differences in predictors of VG training success as well as various outcomes of the training. Complex VGs were found to affect memory, multitasking, spatial rotation, processing speed and emotional skills (Pallavicini et al., 2018). Furthermore, VG training was found to alter the human brain, both its structure and activity (e.g., Palaus et al., 2017; Brilliant et al., 2019). Therefore, such training may strengthen neural scaffolding (Park and Reuter-Lorenz, 2009).

However, some results reported in the literature contradict each other (Boot et al., 2008; van Ravenzwaaij et al., 2014). For instance, it was found that various cognitive faculties in subjects with no prior experience in VG did not improve after VG training (Boot et al., 2008). Researchers also compared expert players with subjects with no VG experience and suggested that either extensive training undergone by experts or baseline individual differences (“self-selection effect”) can lead to professional players performing better in various cognitive tasks (Boot et al., 2008). Therefore, in this review we focus on intervention studies that allow us to make inferences about the causal effect of VG skill acquisition.

Furthermore, individual differences between subjects who may benefit from VG interventions can explain the inconsistent results (Bavelier et al., 2011). Macnamara et al. (2014) estimated that 26% of variability in VG performance is the effect of practicing the game, while the rest is accounted for by the influence of predispositions on neuroanatomical and cognitive performance, and by demographic traits, such as age. We have augmented the STAC model, which explains the relationship between aging and neural mechanisms, to include brain structural predictors of VG training success in young adults and the elderly (Park and Reuter-Lorenz, 2009). We hypothesized that those brain areas which are strongly involved in compensatory mechanisms will be linked to prediction of VG skill performance.

**FIGURE 1:**
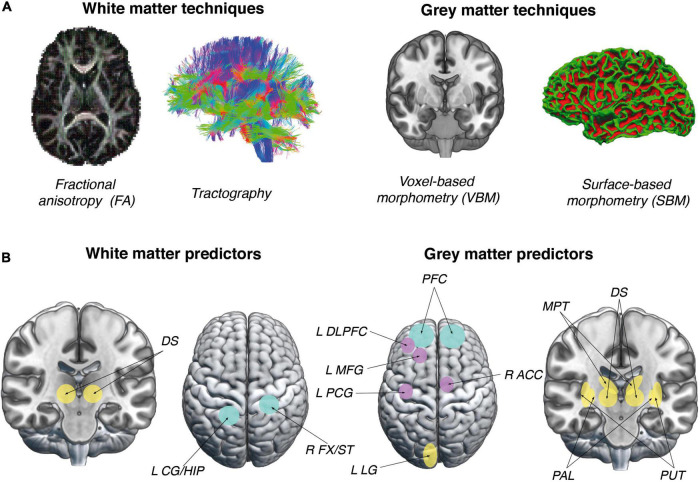
Approaches and predictors used in studies of complex VG skill acquisition. **(A)** Possible approaches to studying neuroanatomical predictors of complex VG skill acquisition. Images on the left represent approaches that can be used to discover WM predictors (FA, Tractography). Images on the right represent approaches that yield GM measures (VBM, SBM). **(B)** Figures in this panel represent significant brain structural predictors of VG complex skill acquisition. Brain regions mostly representative for older adults are marked in purple, younger adults in yellow and both groups in turquoise. Mainly cortical areas predicted VG skill acquisition in the group of older adults and subcortical in the group of younger adults. Figures on the left represent properties of WM in brain areas which were found to be significant predictors of VG performance: DS, dorsal striatum (Vo et al., 2011); L CG/HIP, left cingulum/hippocampus; and R FX/ST, right fornix/stria terminalis (Ray et al., 2017). Figures on the right represent properties of GM in brain areas which were found to be significant predictors of VG performance: DS, dorsal striatum (Erickson et al., 2010); MPT, medial-posterior thalamus (Momi et al., 2019); L LG, left lingual gyrus (Momi et al., 2018); PAL, pallidum; PUT, putamen (Kowalczyk-Grêbska et al., 2021), L DLPFC, left dorsolateral prefrontal cortex; L MFG, left medial frontal gyrus; L PCG, left post central gyrus; R ACC, right anterior cingulate cortex (Basak et al., 2011); and PFC, prefrontal cortex (Head et al., 2002). Brain images were created with MRIcroGL, an open-source 3D-rendering software package (McCausland Center for Brain Imaging, University of South Carolina; https://www.nitrc.org/projects/mricrogl/) and FreeSurfer software (Laboratory for Computational Neuroimaging at the Athinoula A. Martinos Center for Biomedical Imaging; (https://surfer.nmr.mgh.harvard.edu).

In order to understand neuroanatomical factors that predict VG training success, we focused in our review on measures obtained with magnetic resonance imaging (MRI), used to assess the quality of GM (brain morphology) and white matter (integrity of white matter tracts) (see Figure 1A). MRI is commonly used to study the influence of experience on brain structure, due to its high spatial resolution and low invasiveness (Schmidt et al., 2021; for review see Zatorre et al., 2012).

High variability in VG performance outcomes may also be attributed to a misuse of the term “AVG”. This genre combines disparate types of games, which differ in their mechanics, and this makes it difficult to compare different studies. It used to include mainly shooters (first- and third-person, FPS, TPS), but nowadays it also includes a subgenre of strategy games, called Multiplayer Online Battle Arena (MOBA), action real-time strategy (RTS) games, and action role-playing games (RPG). These mixed genres create new methodological challenges for researchers (Dale et al., 2020). Some have already suggested that this stretching of the meaning of the term may explain the inconsistent results in measurements of cognitive performance improvements (Boot et al., 2013). Additionally, inclusion in this category of serious VGs, such as Space Fortress, is problematic (Bediou et al., 2018). In earlier reports, this game was classified as an AVG (e.g., Palaus et al., 2017). Therefore, we decided to take into account the differences between non-commercial and commercial VGs, as proposed by Choi et al. (2020).

A recent review of literature on cognitive enhancement investigated predictors in a wide array of contexts, such as simple laboratory tasks, such as the multitask paradigm (Verghese et al., 2016), complex VG interventions (Erickson et al., 2010; Basak et al., 2011; Ray et al., 2017) and other cognitive training tasks, for instance math training (Supekar et al., 2013), scientific lecture attendance and take-home assignments (de Lange et al., 2016; Baykara et al., 2020). However, as was shown at the beginning of this section, VGs, unlike simple behavioral interventions, allow researchers to investigate real-life skill acquisition processes, and generalize more readily (Boot, 2015; Boot et al., 2017). In the present review – which differs in this respect from similar studies – we discuss extensively the neuroanatomical correlates of complex VG skill acquisition and propose a detailed framework for future research in this area. We also discuss our findings in the light of the STAC. This theory can potentially explain differences in brain predictors of VG skill acquisition between age groups (Park and Reuter-Lorenz, 2009).

Our aim was to shed light on the role of individual differences in complex VG skill acquisition for healthy young and old adults. We have explored the relationship between neuroanatomical predictors and VG learning outcome taking into account the role of neural plasticity after VG training.

## Materials and methods

In preparation for the review, a PRISMA recommendations checklist was followed (Liberati et al., 2009).

### Sources

**FIGURE 2:**
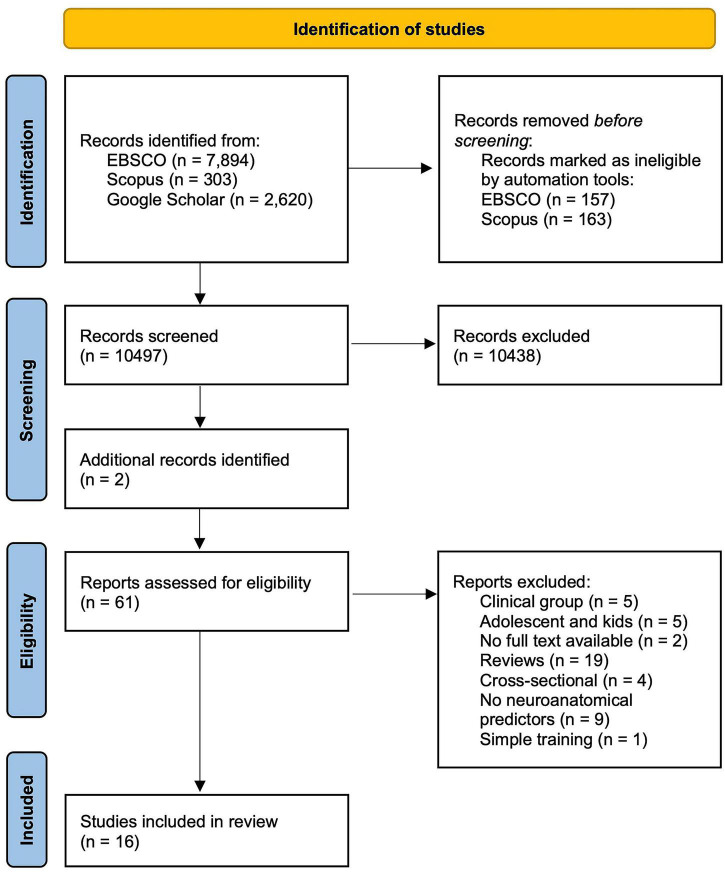
PRISMA Flow diagram for study selection. Source of template: Haddaway et al., 2021.

To select studies for the review, we searched EBSCO, Scorpus and Google Scholar, as well as followed up references given in the articles chosen for analysis. The databases were searched using the following groups of keywords: (1) terms related to VGs: “video game,” “video game training,” “video game skill acquisition,” “complex video game,” “video game playing”; (2) terms related to prediction: “correlation,” “prediction,” “association,” “individual differences”; (3) terms related to neural plasticity: “neural plasticity,” “brain plasticity,” “structural plasticity”; (4) terms related to neuroimaging techniques: “neuroanatomical,” “neural,” “brain,” “MRI,” “SBM,” “VBM,” “DTI,” “connectivity,” “volume,” “structure,” “gray matter,” “white matter.” Only articles in English were included; no limit on date of publication was imposed. The earliest study included in the review was published in 2002 and the most recent one in 2021. Detailed information on database search is presented in Figure 2.

### Inclusion criteria

We limited the scope of our review to studies on healthy adults, young (over 18 years old) and older. We excluded studies on children and adolescents in order to concentrate on predictors of training success independent of developmental processes. We have not included analysis of clinical groups as comparison of the healthy adults to the group with the significant loss of brain functions is a topic for the separate work and will not significantly support our argument of the effect in healthy aging.

Moreover, we concentrated on longitudinal studies of naive participants learning to play a video game they had never played before, practicing for the duration of at least one session. We excluded retrospective studies to minimize the impact of confounding factors on brain structure and to investigate causal relationships between individual differences and VG training benefits. We focused on studies that provide information on the relationship between training success and baseline neuroanatomical measures collected during the first session prior to training. Studies looking for correlates of skill acquisition connected with video games of any genre (including FPS, SVG and its subtype RTS, as well as puzzle) were included in the analysis. According to the suggestion of one of the reviewers, we have also included studies of neural plasticity after VG training.

### Data collection

In total, 16 studies which met the inclusion criteria presented above were found in the literature eight studies exploring the structural predictors (WM and GM) of VG skill acquisition and eight structural plasticity (WM and GM) after VG training (see Figure 2). For an overview of the studies, see Tables 1, 2.

**TABLE 1:** Summary of studies of structural predictors of skill enhancement in VG complex task training.

Authors, year	Type of brain tissue	Age group	Sample size	Demographic characteristics	Game type	Game genre	Game name	Training type	Total training duration	Session duration	Method of analysis	Measure of skill acquisition	Method of statistical analysis	Brain areas	Effect (r/R2)	Strength of relationship (r)
Head et al., 2002	GM	YA and OA	*N* = 68	Age: *M* = 48.88; *SD* = 16.65 (range 22–80 years), gender: 42 F	NCVG	Puzzle	Tower of Hanoi	ST	1 training session (no information about duration)	2 × 2 blocks 45 min apart	VBM	Speed (avg time per move), efficiency of solution in the first trial (measure of early acquisition) and in the second trial (measure of late skill acquisition)	Correlation and hierarchically nested regression	**Early acquisition:** ↓ LPFC with skill acquisition, also (mediated by differences in non-verbal working memory) affects age-related differences in learning. When hypertensive participants removed – no relation. **Late acquisition: ↓** LPFC significant correlation with skill acquisition, but in regression none of brain areas significant.	**Early acquisition:** *r* = –0.28 **Late acquisition:** *r* = –0.39	Early acquisition: r = –0.28 Late acquisition: *r* = –0.39
Erickson et al., 2010	GM	YA	*N* = 36: fixed priority (*N* = 18), variable priority (*N* = 18)	Age: range = 18–28 years, gender: 26 F (variable priority 12 F, fixed priority 14 F)	NCVG	Action	Space Fortress	LT	20 h (10 sessions)	2 h	VBM	Score improvement (total and subscores) for early and late skill acquisition	Prediction (regression)	**Overall early + late acquisition: ↑** DS significant, HIP and VS ns **Early acquisition:** ↑ VS independently of strategy, DS only for variable priority. **Late acquisition:** ↑ DS only for variable priority.	**Overall early + late:** R2 = 0.23 **Early:** VS L (*b* = 0.37) and R (*b* = 0.33) DS for variable priority strategy L (*b* = 0.42) and R (*b* = 0.31) **Late:** DS for variable priority strategy L (*b* = 0.46) and R (*b* = 0.36)	Overall: *r* = 0.12
Basak et al., 2011	GM	OA	*N* = 20	Age: *M* = 70.1, *SD* = 4.8; gender 15 F	CVG	RTS	Rise of Nations	LT	23.5 h (15 sessions, 5 or 6 weeks)	1.5h	VBM	Difference in overall time spent playing the VG, learning rate (or rate of speed-up in power function of each individual’s game time as a function of the number of games played)	Prediction (regression)	↑ L MFG, L PCG, L DLPFC, R ventral ACC, bilateral cerebellar volume with difference in time. ↓ same areas with learning rate.	Adjusted R2 = 0.62	*r* = 0.31
Momi et al., 2018	GM	YA	*N* = 29: experi- mental group (*N* = 17), control group (*N* = 12)	Experimental age: *M* = 23.2; *SD* = 2.5, gender: 9 F Control age: *M* = 25.6; *SD* = 3.1, gender: 6 F	CVG	AVG (FPS)	CS:GO	LT	30 h (4 weeks, 15 sessions)	2 h	SBM (cortical thickness)	Score changes (based on Kill/Death ratio, corrected for difficulty level)	Prediction (regression)	↑ L LG	R2 = 0.34	*r* = 0.17
Momi et al., 2019	GM	YA	*N* = 35: experi- mental group (*N* = 20), control group (*N* = 15)	Experimental age: *M* = 24.2; *SD* = 2.6, gender: 4 F Control age: *M* = 26.6; *SD* = 3.2, gender: 6 F	CVG	AVG (FPS)	CS:GO	LT	30 h (4 weeks, 15 sessions)	2 h	VBM	Score changes (corrected for difficulty level)	Prediction (regression)	↑ bilateral MPT (bilateral Hb, bilateral Li, right CM, right PuA, PuM, parvocellular part of bilateral MD)	R2 = 0.36	r = 0.18
Kowalczyk-Grêbska et al., 2021	GM	YA	*N* = 16	Age: *M* = 22.94; *SD* = 2.11, gender: 11 F	CVG	RTS	StarCraft II	LT	30 h (3–4 weeks)	Minimum 6 h per week, maximum 10 h per week	VBM	Weighted time spent on every level of difficulty	Correlation	↑ LN (PUT and PAL)	L PUT *r* = 0.67 and R PUT *r* = 0.57, L PAL *r* = 0.62 and R PAL *r* = 0.62	L PUT *r* = 0.67 and R PUT *r* = 0.57, L PAL *r* = 0.62 and R PAL *r* = 0.62
Vo et al., 2011	WM & GM	YA	*N* = 34	Age: *M* = 22; *SD* = 3,8, gender: 26 F	NCVG	Action	Space Fortress	LT	20 h (2–8 weeks)	2 h	VBM	Score improvement	Prediction (MVPA)	↑ DS	**WM:** whole DS: *r* = 0.65 Prediction accuracy was significantly higher from anterior (*r* = 0.82) in comparison to posterior (*r* = 0.38 half of L DS **GM:** DS: *r* = 0.38 Better prediction from WM (*r* = 0.65) in comparison to GM (*r* = 0.02)	WM whole DS: *r* = 0.65 GM whole DS: *r* = 0.38
Ray et al., 2017	WM	YA and OA	*N* = 62: YA (*N* = 31) and OA (*N* = 31)	YA age: *M* = 25,84; *SD* = 4,52, gender: 18 F OA age: M = 65,84; SD = 6,77, gender: 19 F	CVG	AVG and SVG	Tank Attack 3D (AVG) Sushi Go Round (SVG)	ST	3 h (27 sessions)	7 min	DTI (FA)	Highest level reached and learning rate	Prediction (regression) and correlation	↑ L CG/HIP with SVG learning and R FX/ST with AVG learning	L CG/HIP: *r* = 0.28 R FX/ST *r* = 0.27	L CG/HIP: *r* = 0.28 R FX/ST *r* = 0.27

GM, gray matter; WM, white matter; YA, younger adults; OA, older adults; F, females; yrs, years; CVG, commercial video game; NCVG, non-commercial video game; RTS, real time strategy; AVG, action video game; SVG, strategy video game; CS:GO, Counter-Strike: Global Offensive; ST, long-term training; LT, long-term training; h, hours; min, minutes; VBM, voxel-based morphometry; SBM, surface-based morphometry; DTI, diffusion tensor imaging; FA, fractional anisotropy; avg, average; MVPA, multi-voxel pattern analysis; ns, not significant; ↓, decrease; ↑, increase; L, left; R, right; LPFC, lateral prefrontal cortex; DS, dorsal striatum; HIP, hippocampus; VS, ventral striatum; MFG, medial frontal gyrus; PCG, post central gyrus; DLPFC, dorsolateral prefrontal cortex; ACC, anterior cingulate cortex; LG, lingual gyrus; MPT, medial-posterior thalamus; Hb, habenular nucleus; Li, limitans nucleus; MD, mediodorsal nucleus; PuM, medial pulvinar; CM, central medial nucleus; PuA, anterior pulvinar; MDpc, mediodorsal nuclei; LN, lenticular nucleus; PUT, putamen; PAL, pallidum; CG, cingulum; FX, fornix; ST, stria terminalis.

**TABLE 2:** Summary of studies of neural plasticity after VG complex task training.

Authors, year	Type of brain tissue	Age group	Sample size	Demographic characteristics	Game type	Game genre	Game name	Training type	Total training duration	Session duration	Method of analysis	Method of statistical analysis	Brain areas
Szabó et al., 2014	GM	YA	*N* = *56*	Age: *M* = 36.8, *SD* = 10.3, gender: 26 F	CVG	3D adventure	Super Mario 64	LT	28 h (8 weeks)	30 min daily	VBM	ANOVA	↑R HIP
Kühn et al., 2014	GM	YA	*N* = 48: experimental (*N* = 23), passive control (*N* = *25*)	Age: M = 24.1, SD = 3.8, gender: 70.8% F; Experimental age: *M* = 23.7, *SD* = 3.0, gender: 17 F; Passive control age *M* = 24.5, *SD* = 4.4, gender: 17 F	CVG	3D adventure	Super Mario 64	LT	28 h (8 weeks)	30 min daily	VBM	ANOVA	↑R HIP, DLPFC and bilateral cerebellum. HIP correlated with changes from egocentric to allocentric navigation strategy
West et al., 2017	GM	OA	*N* = 33: experimental (*N* = 8), active control (*N* = 12), passive control (*N* = 13)	Age: *M* = 69.3, *SD* = 5.7, 55.5% F; Experimental age: *M* = 67.7, *SD* = 4.3, 83.3% F; Active control age *M* = 66.9, *SD* = 3.9, 76.9% F Passive control age *M* = 66.9, *SD* = 3.9, 76.9% Fl	CVG	3D adventure	Super Mario	LT	60 h (24 weeks)	5 days a week for 30 min	VBM	*t*-test, ANOVA	↑HIP, cerebellum pre-post training and significant interaction with group - increase in VG group in comparison to control
Kühn et al., 2018	GM	YA	*N* = 40: training group (*N* = 20), active control (*N* = 20)	age: *M* = 27.4, *SD* = 6.6, gender: 18 F, Experimental age: *M* = 28.8, *SD* = 6.8; Active control age: *M* = 26.1 years, *SD* = 6.4	CVG	3D adventure and 2D adventure	Super Mario 64 (3D); Super Mario Bros (2D)	LT	28 h (8 weeks)	30 min daily	VBM	*t*-test	**3D vs. 2D:** ↑PFC, rACC, and R PrCG, precuneus and L MTG ↓bilateral caudate nucleus
West et al., 2018	GM	YA	*N* = 43	Age: *M* = 23.1, SD = 3.66, gender: 29 F	CVG	Action (FPS), 3D adventure	Different games (Study 2: Call of Duty Modern Warfare 2 and Mario; Study 3: Dead Island; Borderlands 2 and Mario)	LT	90 h (8 weeks)	2–4 h 3 times a week	VBM	*t*-test	**Action:** ↑HIP spatial navigation learners, ↓HIP for non-spatial learners and ↑amygdala. **3D adventure**: ↑HIP and entorhinal cortex
Diarra et al., 2019	GM	OA	*N* = 33: experimental (*N* = 8), active control (*N* = 12), passive control (*N* = 13)	Age: *M* = 69.3, *SD* = 5.7, 55.5% F Experimental age: *M* = 67.7, *SD* = 4.3, 83.3% F; Active control age *M* = 66.9, *SD* = 3.9, 76.9% F Passive control age *M* = 66.9, *SD* = 3.9, 76.9% F	CVG	3D adventure	Super Mario	LT	60 h (24 weeks)	5 days a week for 30 min	VBM	*t*-test, ANOVA	↑FEF pre-post training and significant interaction with group - increase only in VG
Colom et al., 2012	GM and WM	YA	*N* = 20, experimental (*N* = 10), control (*N* = 10)	Age: M = 18.95, SD = 2.65, all F	CVG	Puzzle	Professor Layton and The Pandora’s Box	LT	16 h (4 weeks)	4 h per week	VBM, SBM (cortical thickness and surface area), DTI (FA, AD, RD)	t test	**GM: ↑**PFC and less in parietal and temporal areas. SBM ns. **WM:** AD ↑ HIP and RD ILF. No transfer to intelligence.
Strenziok et al., 2014	WM	OA	*N* = *42*	Brain Fitness age: *M* = 69.70, *SD* = 6.9, gender: 8F; Space Fortress age: *M* = 68.52, *SD* = 5.6, gender: 8F; Rise of Nations age *M* = 69.4, *SD* = 2.3, gender: 9F	CVG and NCVG	AVG, RTS, brain training	Brain Fitness, Space Fortress, Rise of Nations	LT	66 h (6 weeks)	6 days a week for 1 h daily	DTI (FA, AD, RD)	ANOVA	↑AD of L LG, L thalamus in all genres. **Brain training vs. FPS:** ↑ in R TOJ **Brain training vs. RTS:** ↑in POTJ

GM, gray matter; WM, white matter; YA, younger adults; OA, older adults; F, females; yrs, years; CVG, commercial video game; NCVG, non-commercial video game; RTS, real time strategy; AVG, action video game; SVG, strategy video game; ST, long-term training; LT, long-term training; h, hours; min, minutes; VBM, voxel-based morphometry; SBM, surface-based morphometry; DTI, diffusion tensor imaging; FA, fractional anisotropy; AD, axial diffusivity; RD, radial diffusivity; avg, average; ns, not significant, ↓, decrease; ↑, increase; L, left; R, right; HIP, hippocampus; DLPFC, dorsolateral prefrontal cortex; LG, lingual gyrus; ILF, inferior longitudinal fasciculus; rACC, rostral cingulate cortex; MTG, middle temporal gyrus; FEF, frontal eye fields; SPG, superior parietal gyrus; IFG, inferior frontal gyrus; PrCG, precentral gyrus; FG, frontal gyrus; TOJ, temporo-occipital junction; POTJ, parieto-occipito-temporal junction.

We have identified 16 variables: Type of brain tissue (WM, GM, WM, and GM), Age group (younger or older adults), Sample size, Demographic characteristics, Game type (commercial or non-commercial), Game genre (FPS, SVG, RTS, puzzle), Game name, Training type (long or short-term training), Total training duration, Session duration, Technique of analysis (voxel-based morphometry – VBM, surface-based morphometry – SBM, diffusion tensor imaging – DTI), Measure of skill acquisition, Method of statistical analysis (correlation, regression, multi-voxel pattern analysis – MVPA), Brain areas and Effect size (Table 1). We have categorized games into non-commercial or serious games, and commercial VGs, in accordance with a taxonomy recently proposed by Choi et al. (2020), discussed in the preceding section.

## Results

### Sample

We begin by describing sample characteristics for studies. The total sample size of analyzed studies was *N* = 834 (*N* = 519 predictor studies and *N* = 315 structural plasticity).

Most of the studies of predictors were conducted on young subjects (62.5%); only 12.5% included older subjects and 25% compared the two age groups. Proportions were similar for neural plasticity in which 62.5% of studies were on younger participants. The average sample size of prediction studies was *M* = 37.5 (*SD* = 14.59) subjects. The sample size for the structural plasticity was similar *M* = 39.38 (*SD* = 10.88).

### Video game training protocol

In most studies exploring predictors (75%), long-term training interventions were used, in which total training duration was mostly from 20 to 30 h and lasted from 1 to 2 h per session. In one study the total training duration was not reported, and the only information provided was that training consisted of 1 session (Head et al., 2002). In one study both the total duration and the duration of a single session were short (3 h and 7 min, respectively), but the training was intensive (27 sessions) (Ray et al., 2017).

Studies of neural plasticity used only long-term training starting from 16 h. Maximum training duration was 90 h, while the most often participants trained for 28 h.

The majority of predictor and neural plasticity studies used commercially available games (87.5 and 62.5%, respectively). We also observed a trend of increased popularity of commercial VGs after 2011. The genres of commercially available VGs comprised mainly FPS and RTS games for the predictor studies, while in the neural plasticity prevalent was the 3D adventure genre. Only one prediction study compared two games, action and strategy (Ray et al., 2017), whereas all the others limited themselves to just one genre. In case of neural plasticity three studies were conducted comparing different VGs.

Various games were used in the studies under review. In neural plasticity studies Super Mario was used the most often. The two most frequently used games for prediction studies were Space Fortress (non-commercial) and Counter-Strike: Global Offensive (CS:GO) (FPS). It is important to note that, in 2005, Revised Space Fortress was released (Shebilske et al., 2005). Researchers who evaluated this version of the game recommended caution when comparing Space Fortress and Revisited Space Fortress, and suggested that the new version has some advantages over the previous one (Shebilske et al., 2005). In spite of this warning, the studies under analysis fail to state clearly which version of the game was used. Researchers used two games in the strategy genre: Rise of Nations, which includes elements of RTS and Turn-based strategy (TBS), as well as StarCraft II (RTS). One study explored skill acquisition using a traditional puzzle game in the prediction studies (Head et al., 2002). In the neural plasticity studies brain fitness and puzzle games were also explored (Colom et al., 2012; Strenziok et al., 2014).

### Neuroanatomical correlates of performance enhancement following video game training

Researchers studying cortical GM use surface-based and voxel-based representations of brain structure measured with MRI (see Figure 1A). Voxel-based morphometry (VBM) is a technique used to compare volumetric changes in particular regions of interest or in the whole brain (Zatorre et al., 2012). VBM is based on the detection of tissue boundaries in T1 weighted brain images. However, it does not support high sensitivity for specific tissue properties, such as density and size of cells or their myelination (Zatorre et al., 2012). Surface-based morphometry (SBM) is a different method, which can provide insights into cortical thickness, surface area and cortical volume with better differentiation of tissue boundaries (Lemaitre et al., 2012). With SBM, it is possible to estimate the shape of cortical structures, which are represented as triangles (Feczko et al., 2009). In contrast, VBM measures only the density of GM in the brain (Hutton et al., 2008). Previous accounts suggest that VBM and SBM measures can produce inconsistent results, which can be attributed to methodological differences between the two approaches, which make them difficult to compare directly (Hutton et al., 2008).

Yet another method used in studies of complex VG skill learning is the multi-voxel pattern analysis (MVPA). This technique exploits the Support Vector Regression (SVR) machine-learning algorithm to predict participant scores in VGs (Vo et al., 2011).

In order to understand the mechanism of video game learning it seems necessary to focus not only on GM volume and cortical thickness, but also on white matter (WM) microstructure metrics (see Figure 1A). Existing research suggests that, in addition to GM analysis, WM measures are a promising predictor of general learning outcomes (Vo et al., 2011; Ray et al., 2017). Diffusion Tensor Imaging (DTI) is a variant of the diffusion-weighted imaging (DWI) method that makes it possible to observe water diffusion processes in tissues. With it, one is able to map the pathways of nerve fibers, the degree of myelination and the integrity of white matter (Le Bihan et al., 2001). Fractional anisotropy (FA), which measures the degree of anisotropy in the diffusion process, is frequently used to observe properties of white matter. In WM regions, due to the predominance of nerve fibers, the movement of water molecules is restricted by cell membranes or axons covered with myelin (Le Bihan et al., 2001). Therefore, diffusion in WM regions occurs in privileged directions and is anisotropic (Le Bihan et al., 2001).

The majority of studies (75%) analyzed the properties of GM, in order to find the neurocognitive correlates of performance in VG training. Only in one was WM analyzed, and in another both GM and WM were treated as predictors. The VBM approach was used in 75% of the studies, and equal percentages of studies (12.5%) used SBM (cortical thickness) and DTI. As far as statistical analysis is concerned, most researchers focused on one type, namely, regression (50%). Only in one study a classification algorithm, MVPA, was used in order to predict response to training (Vo et al., 2011). Nearly every study adopted a measure of training outcomes different from almost every other. Even though score change was the most popular measure (it was used in four studies), the way it was calculated was different in each case (Erickson et al., 2010; Vo et al., 2011; Momi et al., 2018, 2019).

In almost all studies, neuroanatomical properties measured at the baseline correlated positively with training results; only Head et al. (2002) found a negative correlation.

In order to obtain a unified measure of the strength of the relationship between VG performance and neuroanatomical measures, the standardized coefficient (*r*) was calculated for all studies which used regression analysis.

Its values ranged from *r* = 0.12 for the volume of the dorsal striatum in players of Space Fortress, a non-commercial game (Erickson et al., 2010) to *r* = 0.67 for the volume of the left putamens in players of RTS games (Kowalczyk-Grêbska et al., 2021). For action VGs, the correlation coefficient ranged from *r* = 0.17 (thickness of left lingual gyrus) (Momi et al., 2018) and *r* = 0.18 (volume of bilateral medial-posterior thalamus) (Momi et al., 2019) to *r* = 0.28 (WM of left cingulum/hippocampus) (Ray et al., 2017).

Only in one study a negative correlation was found between the volume of the lateral prefrontal cortex and early and late puzzle game skill acquisition (*r* = –0.39 – late; *r* = –0.28 – early acquisition) (Head et al., 2002).

#### Gray matter

Researchers who studied young adults found that the volume of GM in thalamic nuclei (Momi et al., 2019), in ventral and dorsal striatum (Erickson et al., 2010; Vo et al., 2011) and in lenticular nucleus (Kowalczyk-Grêbska et al., 2021), as well as the thickness of the lingual gyrus (Momi et al., 2018) are significantly correlated with performance enhancement following VG training (see Figure 1B).

Erickson et al. (2010) highlighted an important role played by the ventral striatum in motivation and reward processing during the initial phases of skill acquisition, and of the dorsal striatum in promoting flexibility and procedural learning in its early and late stages, but only in subjects who vary their priority strategy (Erickson et al., 2010). Two groups of participants were trained for 20 h to play Space Fortress, a non-commercial game. The fixed priority group trained overall task performance, whereas the importance of different task components changed through the course of the game for the variable priority group. Interestingly, hippocampus GM was not predictive of performance enhancement in VG training, despite previous reports of its enlargement as the effect of training (Kühn et al., 2014; Szabó et al., 2014).

However, a study by Vo et al. (2011), in which MVPA was used, revealed that WM in the dorsal striatum is, in comparison to GM, a better predictor of training success in subjects learning to play Space Fortress. In this case, the prediction of performance enhancement based on GM in the dorsal striatum was significant, but less accurate in comparison to WM.

Furthermore, a recent study conducted by Kowalczyk-Grêbska et al. (2021) highlighted the importance of GMV of the lenticular nucleus (consisting of the putamen and globus pallidus within the basal ganglia) as a correlate of skill acquisition in StarCraft II, an RTS game. Researchers revealed the relationship between performance in VG training and baseline volume of areas linked to motor, memory, attention and control functions, as well as action selection based on goals (Kowalczyk-Grêbska et al., 2021).

In turn, Momi et al. (2018) showed that the thickness of the left lingual gyrus (which is linked to visuospatial information processing) is a significant predictor of skill acquisition in FPS training. In another study, the same team demonstrated that GM volume of the bilateral medial posterior thalamus can be a significant predictor of VG skill acquisition (Momi et al., 2019). Medial and posterior parts of thalamic nuclei are usually linked to attention and perspective processing (Momi et al., 2019). Participants in both studies played CS:GO, an action FPS, for 30 h.

We now turn to studies exploring GM correlates of skill acquisition in healthy older adults. Basak et al. (2011) has shown that GMV of the left medial frontal gyrus, left post central gyrus, left dorsolateral prefrontal cortex, right ventral ACC and bilateral cerebellar is a predictor of differences in time spent on VG, and is correlated with the learning rate achieved by subjects who played Rise of Nation. The medial prefrontal cortex, which was found to be the strongest predictor of VG skill acquisition by Basak et al. (2011), is linked to motor functions (Chouinard and Paus, 2006), response inhibition and conflict monitoring (Alexander et al., 2007). The post central gyrus or somatosensory area can be involved in feedback mechanisms involving the prefrontal cortex and motor areas, as suggested by Basak et al. (2011), whereas the ACC is known to be involved in errors and conflict detection (Alexander et al., 2007). In turn, the dorsolateral prefrontal cortex is linked to solving conflicts by using attentional control and memory (Carter and van Veen, 2007), executive control (Erickson et al., 2007), and complex skill learning (Kennedy and Raz, 2005). This area was also found to be a significant predictor of training outcomes in a study by Head et al. (2002). The GM volume of the lateral prefrontal cortex, mediated by differences in non-verbal working memory, interacted with differences between young and older subjects in learning the Tower of Hanoi puzzle game, but only at the beginning of a brief period of training. It has also been shown that hypertension may interact with the lateral prefrontal cortex being a predictor of skill acquisition. When hypertensive participants were excluded from the analysis, the relation between the volume of the lateral prefrontal cortex and learning outcomes was no longer statistically significant. Moreover, it has also been revealed that the significance of the volume of the lateral prefrontal cortex as a predictor of skill acquisition diminishes in the later stages of short-term plasticity.

The articles mentioned so far used the VBM and SBM approaches to study GM correlates of VG performance enhancement. In the next section we discuss WM predictors of VG skill acquisition.

#### White matter

White matter analysis is essential if we are to achieve a deeper understanding of neuronal mechanisms in VG skill acquisition. And yet, as far as we know, there have been few studies of brain white matter as a predictor of player performance in video games.

Vo et al. (2011) report that properties of white matter were an accurate predictor of action VG learning success (Space Fortress), and that, within the dorsal striatum, WM voxels were indeed better predictors than GM. Moreover, the anterior half of the left dorsal striatum turned out to be a better predictor than the posterior half (Vo et al., 2011). The authors surmise that long-rage myelinated brain connections are crucial to predicting score enhancement in Space Fortress (Vo et al., 2011). In another study, the authors focused on examining the cognitive and neural bases of two different types of game learning, in order to evaluate their common and specific correlates in the group of young and older adults (Ray et al., 2017). In this study, commercial games were used: Sushi-Go-Round, described as an SVG, and Tank Attack 3D, an AVG. However, Tank Attack 3D has some features that are associated with FPS games. The results show that different WM regions separately predict strategy and action VG learning success. The FA value in the right fornix and stria terminalis area was correlated with action game learning, while the FA value in the medial temporal area (left cingulum and hippocampus) was correlated with strategy game learning; no age differences were found (Ray et al., 2017).

Additionally, one study revealed that higher FA values in the left corticospinal tract at the level of cerebral peduncle predicted faster response times in visual attentional tasks only for VGPs, although WM brain areas are not explicitly mentioned as a predictor of VG performance (Zhang et al., 2015).

The articles under analysis show that there is no consistent pattern of WM properties as a predictor of VG performance (Figure 1B). It is difficult to compare the results, since each article used a different approach to measure white matter microstructure. Therefore, our recommendation for future studies would be to use not only consistent methods of analysis, but also complementary ones, such as structural brain connectivity analysis.

### Neural plasticity after video game training

Studies of neural plasticity after VG training included eight research papers. Similarly to the predictor research, most of them explored the topic of GM (75%). There was only one report on WM and one on both measures. Most researchers used VBM to study GM (75%), while there was only one study using also SBM. For WM DWI was used with measures such as, FA, axial diffusivity (AD) and radial diffusivity (RD).

Research on younger adults revealed that GM in such areas as frontal cortex (PFC, DLPFC, precentral areas), parietal (precuneus), temporal (middle temporal gyrus) and cerebellum increased after the VG training (Colom et al., 2012; Kühn et al., 2014, 2018; Szabó et al., 2014; West et al., 2018). Among the subcortical areas ACC and amygdala were increased after the training (Kühn et al., 2018; West et al., 2018). Most studies were conducted on the 3D platformer game Super Mario, only two included other genres such as puzzle (Colom et al., 2012) and action (West et al., 2018). Areas enlarged after the VG training are linked to spatial navigation, strategic planning, working memory and motor performance (Colom et al., 2012; Kühn et al., 2014, 2018; Szabó et al., 2014; West et al., 2018).

Only in one study on younger adults WM AD of hippocampus and RD of inferior longitudinal fasciculus (occipitotemporal area) increased after the VG training (Colom et al., 2012).

Furthermore, the changes in neural plasticity after training in the group of younger subjects were dependent on the type of navigation strategy in action and adventure games – spatial vs. non-spatial (Kühn et al., 2014; West et al., 2018) as well as VG design (3D vs. 2D) in adventure VGs (Kühn et al., 2018). Allocentric or spatial navigation strategies are linked to hippocampus-dependent processing, whereas egocentric or response navigation to caudate processing. Spatial learning navigation relies on learning the relationship between the landmarks in the environment, whereas response learning strategy is linked to learning actions from various starting points (West et al., 2018). Egocentric strategy can be potentially enhanced in first-person games, whereas allocentric in bird’s-eye perspective games (Kühn and Gallinat, 2014). West et al. (2018) shows that in action VG group GM hippocampal volume was increased for spatial navigation learners, whereas non-spatial learners had GM volume decreased in hippocampus and increased in amygdala. Increase in amygdala GM may be linked to more frequent stress reactions of response learners. Group playing the 3D adventure game had an increase in either hippocampus or entorhinal cortex. Researchers suggested that usage of spatial learning strategies should be encouraged especially in action VG games (for example by designing games without the GPS).

Different results regarding neural plasticity were obtained comparing groups playing games with different mechanics such as Super Mario 64 (3D orientation) and Super Mario Bros (2D - only left to right movements in two dimensions) in the study by Kühn et al. (2018). Significant increase was found in PFC. Other areas found to be significantly enlarged after 2-month training in the 3D group were linked to motor-related processing (rostral ACC and right precentral cortex) as well as multisensory processing (precuneus and left middle temporal gyrus near temporoparietal junction). Decrease in the GM volume of bilateral caudate nucleus was observed in 3D platformer players in comparison to 2D (but significant only with liberal threshold).

Studies additionally explored some important aspects of training such as transfer to intelligence. Colom et al. (2012) shows that even if researchers observe neuroanatomical changes after the training, it does not necessarily link to transfer effects in cognitive tasks as there was no interaction with intelligence test scores improvements. It is worth to note that the research was conducted on the homogeneous group of young females who played puzzle game.

Results for elderly revealed changes in various areas, but similarly to younger adults, they included cortical and subcortical areas. Such areas were in the frontal cortex (precentral gyrus, frontal gyrus, frontal eye field), superior parietal gyrus, cerebellum and HIP (Strenziok et al., 2014; West et al., 2017; Diarra et al., 2019).

It was shown that AD value was increased for subcortical structures such as LG and thalamus for action, RTS and puzzle games (Strenziok et al., 2014). Brain training games were additionally compared to other genres and researchers revealed positive differences between them and FPS in WM of temporo-occipital junction (TOJ) and RTS in WM of parieto-occipito-temporal junction (POTJ) (Strenziok et al., 2014). Heightened WM integrity in the ventral attention network, responsible for the bottom-up reorientation of attention, was linked to the far transfer to the everyday problem solving/reasoning (including interpreting a phone bill rate chart or a prescription label) after brain training games (Strenziok et al., 2014).

**FIGURE 3:**
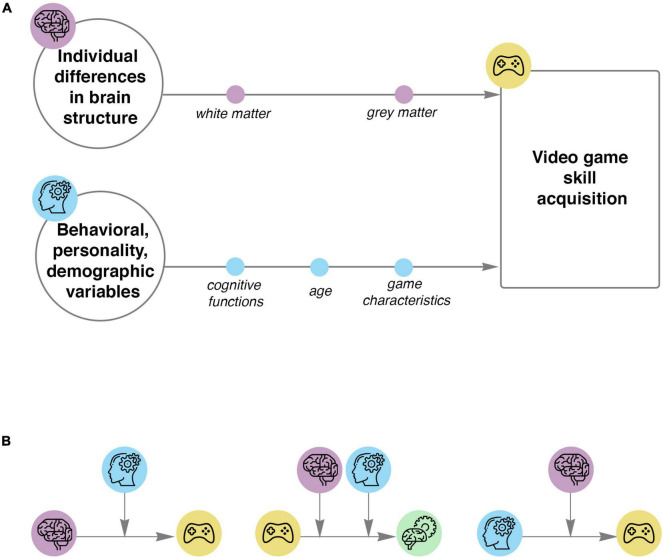
Models to use in studies of complex VG skill acquisition. **(A)** Schemas represent the relationship between baseline characteristics and VG skill acquisition. The first schema represents structural predictors of VG skill acquisition. The second schema shows behavioral, personality, demographic and game predictors of VG performance. **(B)** Proposed complex models of VG skill acquisition using moderation and mediation variables. On the left: baseline structure predicts VG skill acquisition, while behavioral, personality and demographic variables influence the initial relationship. In the middle: relationship between the VG training and neural plasticity, moderated by baseline individual differences in brain structure and behavioral, personality as well as demographic variables. On the right: individual differences in behavioral, personality and demographic characteristics predict VG performance, and baseline structural measures influence the initial relationship. Icons were obtained from Flaticon.com.

In the present review, we have analyzed the reports on empirical studies of structural brain correlates of successful acquisition of complex skills involved in playing video games as well as analysis of neural plasticity after VG training. We chose to focus on VGs, as they are an attractive alternative to traditional computer-based cognitive interventions. We outline statistical models for future studies in the domain of VG complex skill acquisition (Figures 3A,B). Firstly, we suggest that future studies should include both neural and behavioral methods to investigate predictors of VG training outcomes. It is still an open question which structural brain properties are the most accurate predictors of cognitive performance in a game, and therefore, both brain GM and WM properties should be used to shed light on the mechanisms of learning complex skills involved in playing a VG. Secondly, models of VG skill acquisition of greater complexity are needed, incorporating various correlates, as well as mediators and moderators of VG training outcomes. Studies examining the relationship between VG training and neural plasticity after training should also take into account the role of neuroanatomical as well as behavioral factors which can be moderators of the effect of training on plasticity. Thirdly, not only the baseline level of neuroanatomical measures, but also the brain structures during early and late stages of VG training should be used to predict the final outcome of VG training.

## Discussion

Complex training with VG is a cognitive enhancement activity, which can facilitate neuroplasticity and improve mental functions. However, various factors can interact with the effectiveness of this intervention, such as individual differences in brain structure and age.

We found that skill enhancement in young subjects is associated with baseline subcortical brain volume. The volume of GM in the basal ganglia was shown to predict training outcomes in a study that used RTS VGs (Erickson et al., 2010; Kowalczyk-Grêbska et al., 2021); analogous results were obtained for WM voxels in a study with a non-commercial VG (Vo et al., 2011) and for thalamic nuclei volume and lingual gyrus thickness in the case of FPS skill acquisition (Momi et al., 2018, 2019). In studies of older adults, the prefrontal cortex with its different sub-areas, in particular, the medial prefrontal cortex and, to a lesser degree, the dorsolateral prefrontal cortex, turned out to be good predictors of training outcomes in a study using an RTS game (Basak et al., 2011), while the lateral prefrontal cortex played a similar role in a study using a puzzle game (Head et al., 2002).

It is often claimed that cerebral cortex activity underlies many of the higher-level cognitive and executive functions (e.g., decision making, language, motor control) (Bechara et al., 2000; Cadwell et al., 2019), while subcortical structures process more primitive functions (e.g., emotion, pain, reward processes) (Bingel et al., 2002; Hikosaka et al., 2008). However, according to recent reports, subcortical regions not only transmit information to the cerebral cortex, but may also play a role in cognitive, motor and social functions (Kramer et al., 2002; Johnson, 2005; Bickart et al., 2011; Pauli et al., 2016; Koshiyama et al., 2018). What is more, cortical and subcortical areas, such as PFC and basal ganglia, are continually interacting with each other (Hazy et al., 2007; van Schouwenburg et al., 2010).

These results are consistent with the STAC, proposed in 2009 by Park and Reuter-Lorenz and revisited in 2014 (Reuter-Lorenz and Park, 2014). To the best of our knowledge, our work was the first to describe the neuroanatomical predictors of complex skill acquisition (specifically, video game training) in the light of STAC.

### The role of aging in video game skill acquisition and the scaffolding theory of aging and cognition

Park and Reuter-Lorenz (2009) suggested that, with aging, adults face various brain structure changes in GM and WM, which include cortical thinning, volume shrinkage, loss of white matter integrity, increase in WM hyperintensities and dopamine depletion (Gunning-Dixon and Raz, 2003; Morcom et al., 2003; Resnick et al., 2003; Head et al., 2004; Raz et al., 2004; Wen and Sachdev, 2004; Gutchess et al., 2005; Grady et al., 2006; Persson et al., 2007; Zheng et al., 2018). Furthermore, age-related function deterioration is observed, including weaker default mode network suppression, altered frontal activity caused by difficulties in switching from default state into more active modes of cognitive processing and changes in ventral-visual, motor and medial temporal lobe areas (Park and Reuter-Lorenz, 2009). This triggers off compensatory mechanisms – for instance, overactivation of prefrontal areas – recruited to counteract these degenerative processes (Park and Reuter-Lorenz, 2009). The model also suggests that neural scaffolding can be strengthened by learning and cognitive training interventions, such as new, cognitively challenging tasks in enriched environments (Park and Reuter-Lorenz, 2009). As discussed in this review, VGs fulfill the definition of a complex stimulating activity. Brain deterioration, compensatory scaffolding and scaffolding enhancement affect general cognitive functions (Park and Reuter-Lorenz, 2009).

**FIGURE 4:**
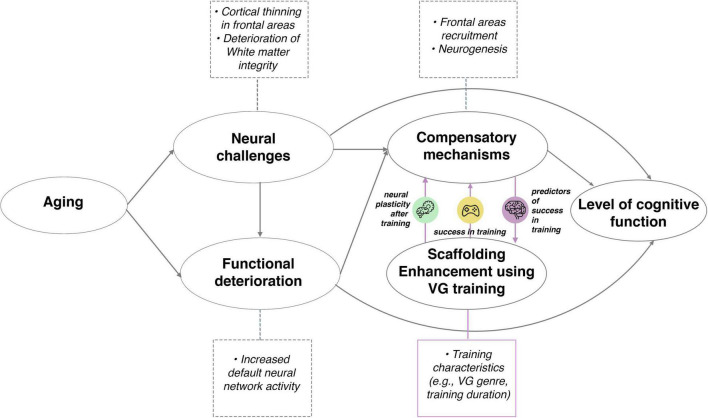
Relationship between aging, brain structural predictors of VG skill acquisition and neural plasticity after training in the context of the Scaffolding Theory of Aging and Cognition (adapted from Park and Reuter-Lorenz, 2009). The model is extended by the addition of predictors of success in scaffolding enhancement activities (VG training) (marked by purple circle “predictors of success in training,” green circle “neural plasticity after training” and yellow circle “success in training”). Neural and functional challenges appear with aging, which are compensated for by various biological mechanisms. Scaffolding enhancement activities, such as complex, challenging VG training, can improve the compensatory mechanisms, which influence general cognitive functioning as they change with age. Success in VG used as an enhancement activity can be predicted from mainly cortical areas in older and subcortical areas in younger adults. Among the older subjects, those whose compensatory mechanisms in the frontal cortical areas are more effective, perform better in VG training. Instead of the subcortical areas usually linked to the success of VG training in younger adults, the elderly engage cortical structures to the greater extent to successfully perform the task. The “success in training” circle indicates the relationship between the behavioral outcomes of training and compensatory mechanisms as well as later cognitive functioning of people, while “neural plasticity after training” circle indicates the relationship between the training and neural plasticity, which are also linked to compensatory mechanisms and later level of cognitive functioning. Training features (e.g., genre, training duration and frequency) can also interact with the relationship between brain predictors, performance in training and neural plasticity (the purple square). Icons were obtained from Flaticon.com.

The present review makes a novel contribution by discussing the role of brain structural predictors of scaffolding enhancement intervention in aging and their bilateral relationship with compensatory mechanisms, presented in Figure 4 (adapted from Park and Reuter-Lorenz, 2009).

Researchers studying the ways in which the brain changes with age found that the frontal and temporal lobes suffered the sharpest decrease in cortical thickness, while the occipital lobe was the least affected (Zheng et al., 2018). Others have also observed a decrease in both GM and WM volume in these brain areas in adults over 59 years of age (Resnick et al., 2003; Raz et al., 2004). Interestingly, increased frontal lobe activation (Morcom et al., 2003; Gutchess et al., 2005) and reduced suppression of the default network (Grady et al., 2006; Persson et al., 2007) was also observed to come with age. According to the STAC model, these are compensatory mechanisms to offset the above-mentioned deterioration. Therefore, one should expect mostly frontal areas – such as the medial prefrontal cortex, the dorsolateral prefrontal cortex and the lateral prefrontal cortex – to predict VG skill acquisition in older subjects, given the role these areas play in compensatory mechanisms. It appears that such an effect can indeed be observed in older subjects who are learning to play games, regardless of game genre (RTS in Basak et al., 2008) or puzzle in Head et al. (2002).

However, even though GM volume in subcortical areas also decreases with age, this relationship is not as consistent as in the case of the cerebral cortex (Zheng et al., 2018). The volume of GM in the thalamus decreases with age, but in the hippocampus this relationship is non-linear, with an initial increase, followed by a decrease around the age of 50 (Zheng et al., 2018). Interestingly, this area was found to be among those in which age-related volume decrease is the sharpest (Raz et al., 2004) – and yet, as shown in our review, it is not a significant predictor of VG skill acquisition in older subjects. Furthermore, no significant relation was found between age and volumes of the putamen, pallidum, accumbens area, amygdala and caudate (Zheng et al., 2018), though Raz et al. (2004) did find an age-related caudate volume reduction. Other areas that are relatively unaffected by age include the primary visual cortex and the entorhinal cortex (Raz et al., 2004). Indeed, these areas are not significantly predictive of VG skill acquisition in older subjects.

Together with GM volume reduction, the structural integrity of WM also deteriorates, and other degenerative processes, such as axonal shrinkage and demyelination, accompany aging (Gunning-Dixon and Raz, 2003). WM integrity is affected and WM hyperintensities appear in frontal regions of the brain (Head et al., 2004; Wen and Sachdev, 2004), which can be linked to slower information processing and behavioral changes (Park and Reuter-Lorenz, 2009). However, the study that analyzed predictors of VG training success in different age groups by measuring WM found no significant difference between participants (Ray et al., 2017). Studies with young participants have shown that WM volume in the dorsal striatum (Vo et al., 2011), and WM integrity (FA value) in the right fornix/stria terminalis and medial temporal area (left cingulum/hippocampus) (Ray et al., 2017) are significant predictors of success in games. The limited findings that are available do not provide enough information to draw clear conclusions concerning the role of age-related WM compensatory mechanisms in the prediction of VG training success.

In summary, frontal cortical areas deteriorate with age and exhibit compensatory mechanisms, such as frontal recruitment (Park and Reuter-Lorenz, 2009). As the authors of the STAC model suggest, the prefrontal cortex is responsible for scaffolding processes that accompany aging. In the context of VG skill acquisition, individual differences in such areas as the medial prefrontal, dorsolateral prefrontal and lateral prefrontal cortex are the most important predictors of VG complex skill learning in older subjects.

The higher the occurrence of compensatory scaffolding in the frontal areas, the better older participants are at VG training; therefore, these regions are predictive of VG training success, as shown in our review. Elderly engage cortical structures instead of subcortical areas to the greater extent to successfully perform the task in contrast to the group of younger subjects. However, in the case of neural plasticity, there is no such relationship - in both groups there are changes after VG training in cortical (especially frontal) and subcortical (especially hippocampal) areas. Structural brain predispositions to perform well in VG training should not necessarily overlap with the brain areas in which neural plasticity occurs as a result of the VG training but more research is needed as the interaction of them seems to be the most probable.

### The role of other factors in the study of structural predictors of video game skill acquisition

Only one study compared two types of games, action and strategy (Ray et al., 2017) and revealed differences in predictors of skill acquisition by players of strategy games (left cingulum/hippocampus) and action games (right fornix/stria terminalis). In all the other studies only one game genre was used. The significant role of VG genre has already been confirmed through the analysis of differences in cognitive improvement after VG training (Oei and Patterson, 2013; Dobrowolski et al., 2015).

It is unfortunate that no consistent taxonomy of VG genres has been adopted by researchers. A good illustration of this point is provided by Space Fortress which some researchers classify it as an AVG (Palaus et al., 2017), while others reject this classification due to differences in game mechanics (Bediou et al., 2018). We believe it is very important to distinguish between commercial and non-commercial VGs, as recently proposed by Choi et al. (2020).

In the studies covered in our review, training duration ranged from one session, consisting only of four blocks, to 30 h. In most of the studies (75%), long-term interventions, lasting for 20—30 h, were used. Therefore, it is difficult to draw any conclusions regarding short-term plasticity during VG training. It was reported previously that even a short time span, such as less than 1 h, can lead to skill enhancement (Head et al., 2002; Hofstetter et al., 2013; Ray et al., 2017). Only one study explored the role of GMV of different subcortical brain areas during early and late stages of complex VG skill acquisition. Individual differences in the volume of GM in the ventral striatum selectively predicted performance enhancement in the early stages of learning, while the dorsal striatum played the same role in both the early and late stages (Erickson et al., 2010). This indicates the need for further exploration of various structural predictors of early – as contrasted with late – stages of learning.

Voxel-based morphometry studies revealed that individual differences in the volume of the ventral and dorsal striatum of the basal ganglia (Erickson et al., 2010), the lenticular nucleus within the basal ganglia (Kowalczyk-Grêbska et al., 2021), the medial-posterior thalamus (Momi et al., 2019) and the prefrontal cortex (Head et al., 2002; Basak et al., 2011) are associated with performance enhancement in VG training. In contrast, an SBM study has shown that lingual gyrus thickness is a significant predictor of performance enhancement in VGs (Momi et al., 2018). Due to differences in methodology and the unequal number of studies that use these two methods, it is difficult to compare their findings directly. Most of the studies used VBM (75%), which is a function of two relatively uncorrelated measures, such as cortical thickness and cortical surface area (Hutton et al., 2008). In the context of GM studies, these two newly developed measures should be more widely used, as they are more sensitive to tissue boundaries. To study plasticity during learning, experiments could take advantage of both VBM and SBM (as complementary approaches) to establish reliable conclusions concerning complex VG skill acquisition.

### A framework for the study of video game skill acquisition predictors

We have identified several potential improvements in the analysis of already acquired data and in the design of new studies. Research has already shown the relevance of game genres for the enhancement of cognitive abilities (Oei and Patterson, 2013; Dobrowolski et al., 2015; Green et al., 2017). Future studies with VGs should take into account various aspects of the games, such as variable priorities, and should also use longer training sessions (longitudinal studies). What is more, the vast majority of studies used measures of skill acquisition incompatible with those in any other study, including: score or rate of improvement, initial score (Erickson et al., 2010; Vo et al., 2011; Hofstetter et al., 2013; Momi et al., 2018, 2019), learning rate based on power calculations, or even measures based on time spent per move or total playing time (Head et al., 2002; Basak et al., 2011). In future research, the ways in which various skill acquisition measures influence the results of prediction models should be explored.

There is a growing need for more complex models of VG skill acquisition, which take into account individual differences and task-related predictors, mediators and moderators of the effect (Figure 3B). Head et al. (2002) adopted a more complex approach, which allowed them to show that the volume of the lateral prefrontal cortex interacts with age-related differences in learning VG, mediated by working memory capacity. Though statistically significant in the population of healthy participants, this relation is not significant in the population of hypertensive subjects, which suggests that biological factors may play a role in this effect. Behavioral moderators should also be studied, because such variables as the strategy adopted by the participant can also influence which areas are predictive of training success (Erickson et al., 2010). We suggest that potential moderators of the relationship between baseline neuroanatomy and training outcome may include the desire to play, stress events and inflammation (Szabó et al., 2014), game genre and mechanics (Kühn et al., 2018). We base our conclusion on the fact that these variables were previously shown to influence brain plasticity after VG intervention. There are no studies examining the relationship between neuroanatomical predictors and neural plasticity after VG training which can shed light on the role of different brain areas for complex skill acquisition.

What is more, longitudinal studies analyzing neuroanatomical measures obtained not only before training, but also immediately after, as well as in follow-up studies, can be a valuable source of information on moderators of the relationship between baseline brain structure and training performance.

Based on our analysis, we propose a framework for studies of predictors of VGs complex skill acquisition (Figure 3). Despite higher costs and challenging methodology, this approach would allow researchers to gain a deeper understanding of this phenomenon. Prediction models of greater complexity should be developed, in order to adjust training regimes to individual abilities and to make them more engaging, challenging and motivating for a given person. Preliminary analysis suggests that the correlates of VG skill acquisition depend significantly on age. The STAC framework may be able to explain this dependence, the relationship between structural predictors of VG skill acquisition and age has not been sufficiently studied to warrant any strong belief in this conclusion.

In summary, the present review highlights several insights concerning structural brain correlates of game learning success, which can be useful in optimizing training for healthy older and younger adults and in developing regimes and selection procedures for professional players. We propose a framework for future studies of complex skill acquisition using VGs. Complex VGs offer an attractive, accessible and research-based training tool, which creates opportunities for practical applications of the findings of this review concerning cognitive enhancement and empirical investigation of complex skill acquisition. We have found that the brain structures which predict VG skill acquisition are different in young and older subjects. This observation can be further studied with the help of the STAC framework. Based on our review, we created a framework for studying predictors of VG complex skill acquisition. The framework may lead to improvements in the effectiveness of interventions and in the methodology of future cognitive training studies.

## Author contributions

AK and PL: conceptualization, visualization, and writing – original draft and review and editing. AB: supervision, funding acquisition, and writing – review and editing. NK-G: conceptualization, supervision, and writing – review and editing. All authors contributed to the article and approved the submitted version.

## Conflict of interest

The authors declare that the research was conducted in the absence of any commercial or financial relationships that could be construed as a potential conflict of interest.

## Publisher’s note

All claims expressed in this article are solely those of the authors and do not necessarily represent those of their affiliated organizations, or those of the publisher, the editors and the reviewers. Any product that may be evaluated in this article, or claim that may be made by its manufacturer, is not guaranteed or endorsed by the publisher.

## Abbreviations

VG, VGs: video game(s)
GM: gray matter
WM: white matter
LG: lingual gyrus
ILF: inferior longitudinal fasciculus
AVG: AVGs – action video game (s)
FPS: first-person shooter
TPS: third-person shooter
MOBA: Multiplayer Online Battle Arena
RTS: real-time strategy
RPG: role-playing game
SVG, SVGs: strategy video game (s)
AI: artificial intelligence
TG, TGs: traditional game (s)
SG, SGs: simulation game (s)
MRI: magnetic resonance imaging
VBM: voxel-based morphometry
SBM: surface-based morphometry
DTI: diffusion tensor imaging
MVPA: multi-voxel pattern analysis
CS:GO: Counter-Strike: Global Offensive
SVR: support vector regression
FA: fractional anisotropy
AD: axial diffusivity
RD: radial diffusivity
LG: lingual gyrus
MPT: medial-posterior thalamus
LPFC: lateral prefrontal cortex
LN: lenticular nucleus
DS: dorsal striatum
VS: ventral striatum
GP: globus pallidus
PuM: medial pulvinar area
MDpc: mediodorsal nuclei
MFG: medial frontal gyrus
PCG: post central gyrus
DLPFC: dorsolateral prefrontal cortex
ACC: anterior cingulate cortex
MPFC: medial prefrontal cortex
CST: corticospinal tract
PFC: prefrontal cortex
IFG: inferior frontal gyrus
PAL: pallidum
PUT: putamen
CG: cingulum
HIP: hippocampus
FX: fornix
ST: stria terminalis
YA: younger adults
OA: older adults
F: females
yrs: years
CVG: commercial video game
NCVG: non-commercial video game
ST: long-term training
LT: long-term training
h: hours
min: minutes
avg: average
ns: not significant
L: left
R: right
rACC: rostral cingulate cortex
MTG: middle temporal gyrus
FEF: frontal eye fields
SPG: superior parietal gyrus
IFG: inferior frontal gyrus
PrCG: precentral gyrus
FG: frontal gyrus
TOJ: temporo-occipital junction
POTJ: parieto-occipito-temporal junction.
